# La precariedad laboral y las limitaciones funcionales: un análisis transversal para la población mexicana

**DOI:** 10.1590/0102-311XES102124

**Published:** 2025-07-04

**Authors:** Daniel Alejandro Márquez-Jiménez, Karla Moreno-Tamayo

**Affiliations:** 1 Universidad Autónoma del Estado de México, Nezahualcóyotl, México.; 2 Centro Médico Nacional Siglo XXI, Instituto Mexicano del Seguro Social, Ciudad de México, México.

**Keywords:** Empleo Precario, Modelo Estadísticos, Factores Sociodemográficos, Precarious Employment, Statistical Models, Sociodemographic Factors, Emprego Precário, Modelos Estatísticos, Fatores Sociodemográficos

## Abstract

El objetivo del trabajo es analizar el vínculo entre la precariedad laboral y la presencia y número de limitaciones funcionales, controlando por variables sociodemográficas con información de la base de datos de la *Encuesta Nacional de Salud y Nutrición* de 2018. El presente es un estudio transversal de base poblacional que utiliza un modelo *hurdle* lineal que está ajustado a través de estimaciones de errores estándar robustos. Se construyó una variable de tipo conteo que mide el número de limitaciones funcionales que tiene cada persona, mientras que la principal variable independiente es un índice sumatorio que clasifica en cuatro rubros al nivel de precariedad laboral del empleo actual. Los resultados muestran que el nivel de precariedad laboral tiene asociaciones significativas en la probabilidad de presentar al menos una limitación funcional, y también para el aumento en el número de limitaciones funcionales, y esa asociación se mantiene aun controlando por las variables teóricamente relevantes como el sexo, la edad y el estado civil.

## Abstract (en)

This study aims to analyze the relationship between precarious employment and the presence and number of functional limitations controlled by sociodemographic variables based on data from the 2018 *Mexican National Health and Nutrition Survey*. This is a population-based cross-sectional study using the linear hurdle model adjusted by robust standard error estimates. A count variable was constructed to measure the number of functional limitations of each person, while the main independent variable is a summative index that classifies unsafety in the current job into four items. The results show that the level of unsafe labor conditions has significant associations with the probability of having at least one functional limitation and also with the increase in the number of functional limitations, and this association is still maintained with the control for theoretically relevant variables such as gender, age and marital status.

## Resumo

O objetivo deste estudo é analisar a relação entre a insegurança no trabalho e a presença e número de limitações funcionais controladas por variáveis sociodemográficas a partir de dados da *Pesquisa Nacional de Saúde e Nutrição* de 2018. Trata-se de um estudo transversal de base populacional utilizando o modelo linear *hurdle* ajustado por meio de estimativas robustas do erro-padrão. Uma variável do tipo contagem foi construída para medir o número de limitações funcionais de cada pessoa, enquanto a principal variável independente é um índice somativo que classifica o nível de insegurança no trabalho atual em quatro itens. Os resultados mostram que o nível de insegurança no trabalho tem associações significativas com a probabilidade de apresentar pelo menos uma limitação funcional e também com o aumento do número de limitações funcionais, e essa associação ainda é mantida com o controle por variáveis teoricamente relevantes como sexo, idade e estado civil.

## Introducción

El trabajo es un elemento central en la vida de las personas, y se ha mostrado fundamental en el desarrollo de enfermedades crónicas ^1^, además de que la propia naturaleza del trabajo tiene implicaciones en las limitaciones de movilidad, sobre todo entre los adultos mayores ^2^. Es importante conocer el impacto que tienen las características de los trabajos en el estado de salud de las personas, sobre todo porque, durante los últimos años, han sido definidas como inestables y precarias ^3^.

Con la llegada de las nuevas tecnologías y la globalización, el mercado laboral general sufrió de un proceso de heterogeneidad, que ha estado caracterizado por la incertidumbre y la vulnerabilidad ^3^ ^,^ ^4^ ^,^ ^5^. En este sentido, los cambios en las dinámicas laborales han hecho que el trabajo al que la mayoría de las personas pueda acceder sea calificado como atípico, temporal, informal o precario ^6^ ^,^ ^7^. Aunque el concepto de trabajo precario ha ganado relevancia desde hace algunos años, y es ampliamente usado, los expertos siguen debatiendo las características específicas que hacen a ciertos empleos ser categorizados de esa manera. 

El concepto de precariedad laboral condensa las condiciones laborales generales que varían entre integración, vulnerabilidad y exclusión ^4^, que además cuenta con dimensiones objetivas y subjetivas, lo que ha permitido analizar el trabajo precario como un espectro que varía en modalidades, e intensidades ^6^. Varias aproximaciones han propuesto soluciones a la medición de la precariedad, y en general todas se articulan a través de cuatro dimensiones fundamentales, representadas en la Figura S1 del Material Suplementario (https://cadernos.ensp.fiocruz.br/static//arquivo/suppl-e00102124_8865.pdf) ^5^ ^,^ ^6^ ^,^ ^7^ ^,^ ^8^ ^,^ ^9^ ^,^ ^10^ ^,^ ^11^ ^,^ ^12^. Algunas investigaciones consideran innecesario ponderar los indicadores de precariedad, debido a que cada indicador es un derecho laboral fundamental que no puede ser reducido frente a otros ^11^ ^,^ ^12^, mientras que otros consideran que hay ciertas ventajas en calcular un indicador sintético ^6^ ^,^ ^7^. 

Una serie de trabajos han puesto énfasis en analizar las asociaciones que tienen las condiciones de trabajo con la salud física y mental de las personas ^2^ ^,^ ^13^ ^,^ ^14^ ^,^ ^15^ ^,^ ^16^, en particular se ha encontrado una asociación negativa entre la precariedad del trabajo con el estado de salud general, y con la aparición de enfermedades crónico-degenerativas en particular ^1^ ^,^ ^15^ ^,^ ^17^ ^,^ ^18^. No obstante, a pesar de que la asociación entre las condiciones de trabajo precario y la salud han sido exploradas con anterioridad ^19^ ^,^ ^20^, hay pocas investigaciones que prioricen el análisis entre el nivel de precariedad del trabajo y las limitaciones funcionales, en sus tres componentes esenciales (funcionamiento, ejecución o participación) ^16^ ^,^ ^17^ ^,^ ^21^ ^,^ ^22^.

Diferentes investigaciones internacionales coinciden en que la situación de vulnerabilidad económica y social derivada de las condiciones de precariedad laboral tienen implicaciones negativas en la salud funcional. Algunos resultados sugieren que hay asociación entre las limitaciones funcionales (limitaciones de movimiento, limitaciones en actividades del día a día), el número de horas dedicadas al trabajo y los salarios bajos, lo que lleva a las personas a situaciones de fatiga y disminución del bienestar ^16^. Por otro lado, estas asociaciones han mostrado estar relacionadas con la edad para el retiro, los fondos de pensiones y las condiciones actuales de los diferentes mercados laborales ^13^ ^,^ ^17^. Por lo tanto, el objetivo de este estudio fue analizar la relación entre precariedad laboral y la presencia y número de limitaciones funcionales con base en una encuesta nacional de salud en México.

## Metodología

### Datos

Estudio transversal de base poblacional con datos de la *Encuesta Nacional de Salud y Nutrición* (ENSANUT) de 2018. ENSANUT tiene representatividad nacional y es elaborada por el Instituto Nacional de Estadística y Geografía y el Instituto Nacional de Salud Pública. La selección de la muestra que ocupa la encuesta se elige con base en la muestra maestra del Marco Nacional de Viviendas 2012, derivado a su vez del Censo de Población y Vivienda de 2010. ENSANUT recolecta temas relacionados con la vivienda, los integrantes del hogar, su salud y nutrición.

La ENSANUT cuenta con un tamaño de muestra de 50.654 viviendas que se distribuyen a lo largo de todas las entidades federativas del país ^23^. La población de estudio se encuentra descripta en profundidad en la Figura S2 del Material Suplementario (https://cadernos.ensp.fiocruz.br/static//arquivo/suppl-e00102124_8865.pdf). Para este análisis, se consideraron únicamente a las personas de 12 años y más (N = 101,076,892), debido a que las preguntas sobre actividad laboral solamente consideran a esa población. A pesar de que solo el 53,8% son personas que declararon haber trabajado durante la última semana antes del levantamiento de la información, se tomó la decisión de integrar 4.015 observaciones a la muestra, porque, aunque respondieron no haber trabajado la semana pasada, declararon tener trabajo, así sea sin pago, en un negocio familiar, en labores del campo o actividades sin especificar. 

Lamentablemente, no todas las personas cuentan con información completa sobre las características de sus trabajos, por lo que la población de análisis se redujo hasta el 40,1% del total de las personas de 12 años o más, lo que es una proporción similar a otras investigaciones sobre precariedad ^24^. Cabe destacar que el salario mínimo es el indicador laboral con la mayor cantidad de observaciones perdidas. 

### Funcionalidad-discapacidad

La ENSANUT cuenta con ocho indicadores de funcionalidad, basados en ocho dimensiones: (1) caminar; (2) ver; (3) mover o usar brazos o manos; (4) aprender, recordar o concentrarse; (5) escuchar; (6) bañarse, vestirse o comer; (7) hablar o comunicarse; (8) realizar sus actividades cotidianas por problemas emocionales o mentales. Cada pregunta evalúa el nivel de dificultad que tienen las personas de acuerdo con la función biopsicosocial que mide, por lo que las respuestas pueden adquirir uno de cuatro valores posibles: 1 = no puede hacerlo; 2 = lo hace con mucha dificultad; 3 = lo hace con poca dificultad; 4 = no tiene dificultad. 

Con la finalidad de analizar el nivel de discapacidades que presenta una persona, se ocupó una clasificación ^2^ que puede tomar uno de tres valores (0 = no tiene dificultad en hacer la actividad; 1 = dificultad; y 2 = le es imposible realizar la actividad). Posteriormente, se sumaron los puntajes de cada dimensión para obtener una escala de hasta 16 puntos que dependen del nivel de dificultad para hacer una o varias actividades. 

### Precariedad laboral

Para la construcción del indicador de precariedad se utilizaron tres de las cuatro dimensiones teóricas propuestas en el marco conceptual (desprotección de las condiciones de trabajo y de su reproducción; inseguridad de los ingresos; y la desregulación de la jornada de trabajo) ^6^. Lamentablemente, la ENSANUT 2018 no cuenta con una pregunta que permita conocer el tipo de contrato bajo el que trabajan, por lo que el nivel de seguridad/inseguridad laboral está subrepresentado.

Se tomaron en total doce indicadores para medir la precariedad laboral, que se pueden ver en la Figura S3 del Material Suplementario (https://cadernos.ensp.fiocruz.br/static//arquivo/suppl-e00102124_8865.pdf). Cada uno de los indicadores se recodificó de manera dicotómica, tomando el valor de 1 ante la ausencia de condiciones favorables en el trabajo, salario menor a dos salarios mínimos y jornadas de trabajo desreguladas (> 48h o < 35h semanales). La definición operacional de los indicadores específicos sobre la vulnerabilidad de las condiciones de trabajo se puede encontrar en la Tabla S1 del Material Suplementario (https://cadernos.ensp.fiocruz.br/static//arquivo/suppl-e00102124_8865.pdf).

Posteriormente, se creó un índice sumatorio simple que permitiera conocer el nivel de precariedad de los trabajadores de la muestra. Se considera que el índice sumatorio simple es la mejor manera de aproximarse a las características del trabajo, debido a que cada uno de los indicadores representan derechos laborales que no pueden ser reducidos frente a otros, por lo que deben de ser considerados con igual nivel de importância ^6^ ^,^ ^7^. Una vez calculado el índice, se agruparon el número de vulnerabilidades presentes en sus condiciones de trabajo en cuatro grupos posibles ^24^ ^,^ ^25^ ^,^ ^26^: sin precariedad (0 = 0 indicadores); precariedad baja (1 = 1 a 6 indicadores); precariedad media (2 = 7 a 10 indicadores); precariedad alta (3 = 11 y 12 indicadores). 

### Características sociodemográficas

Las variables de control estadístico que se usaron fueron: sexo (hombres; mujeres); grupos de edad (12-29; 30-59; ≥ 60); nivel educativo (sin estudios; educación básica [1-9 años]; educación media superior [10-12 años]; superior y más [15 años y más]); estado civil (unido; separado; soltero); índice de activos en el hogar (predicción lineal de una matriz tetracórica de activos en el hogar. Un factor; KMO = 0,85); tipo de arreglo residencial (unipersonal, nuclear, extendida y corresidencia).

### Análisis estadístico

El análisis descriptivo se llevó a cabo a través de frecuencias y proporciones en el caso de variables cualitativas, y medias (desviación estándar − DE) en el caso de las variables cuantitativas. Por otro lado, para conocer la dirección y fuerza de la relación entre las variables se empleó un modelo *hurdle* de dos etapas que permite conocer la influencia que tienen las variables sociodemográficas en la propensión de presentar al menos 1 discapacidad (componente binario) y posteriormente en el cambio unitario de presentar 2 o más problemas de funcionalidad (componente condicional). Para conocer la relación entre las características, laborales, sociodemográficas y de limitaciones, se calculó una serie de modelos a través de la integración sucesiva de variables.

Al analizar un fenómeno como las limitaciones funcionales cuya prevalencia es baja en la población mexicana, se enfrentan varios desafíos metodológicos, sobre todo porque hay pocas personas que presentan alguna de ellas. Hay varias alternativas metodológicas a través de las que se pueden modelar las distribuciones de conteo con varios ceros, en general, se utilizan modelos “cero inflados” que tienen la ventaja de mejorar la bondad de ajuste en comparación con un modelo de conteo tradicional como el Poisson. 

La decisión de utilizar un modelo *hurdle* frente a alternativas como el Poisson zero inflado o una regresión binomial negativa responde principalmente a un criterio metodológico, debido a que el diseño del instrumento asume que todos los ceros (las personas que no presentan ningún tipo de limitación) provienen de una misma fuente estructural ^27^, es decir que la sobre dispersión de ceros proviene de personas sin limitaciones reales (*structural zeros*) y no de aquellas que no reportan tenerlas (*sampling zeros*) ^28^. Los modelos de conteo muestran un pobre ajuste en presencia de grandes cantidades de ceros, mientras que los modelos “inflados” aunque tienen mejor ajuste, consideran que los ceros en la distribución son de dos fuentes distintas ^27^ ^,^ ^28^ ^,^ ^29^. Así, el modelo *hurdle* lo hace apropiado para la investigación, ya que, además de mejorar la interpretación de la relación entre variables ^2^, ha sido usado para analizar fenómenos de acceso a los servicios de salud, y desenlaces en salud con anterioridad ^2^ ^,^ ^28^.

A pesar de que algunas variables sociodemográficas como: activos en el hogar y tipo de arreglo residencial tienen un nivel de medición distinto al individual, se intentó calcular un modelo *hurdle* multinivel para corregir los coeficientes, no obstante, esto complejizó innecesariamente el cálculo del modelo, además de dificultar aún más el uso de los ponderadores ^30^.

Dado que el propósito del análisis es utilizar estas variables únicamente como control estadístico, no consideramos necesario el uso de un modelo *hurdle* multinivel, por el contrario, podría llevar a un modelo menos parsimonioso y que sea más difícil de interpretar. Asimismo, en estrictos términos teóricos es imposible separar las experiencias y características de los hogares con respecto del de los individuos, lo que puede llevar a una distinción analítica artificial ^31^. 

Debido a que la muestra de la ENSANUT 2018 es compleja, todos los cálculos de resultados se hicieron a través del comando “*svy*” del software Stata versión 18 (https://www.stata.com). Esto permite tener una mejor estimación, sobre todo, de los errores estándar. 

### Consideraciones éticas

Este trabajo es un análisis secundario de la base de datos de ENSANUT 2018. Más información sobre ENSANUT 2018 en la página web: https://www.inegi.org.mx/programas/ensanut/2018/#documentacion.

## Resultados

La Tabla 1 presenta las variables incluidas en el análisis con la finalidad de conocer la forma en que se encuentra distribuida la información. Es interesante que en general más del 50% de la población presenta un alto nivel de precariedad laboral, principalmente porque dentro del análisis se integraron a aquellas personas que desempeñan trabajos en sectores informales o tradicionalmente marginados como el campo. Por otro lado, la mayor cantidad de informantes son hombres en edad productiva de 30 a 59 años, además de que más de la mitad están unidos (61,3%). 

**Tabla 1:** Características descriptivas de la población de estudio. *Encuesta Nacional de Salud y Nutrición* (ENSANUT), México, 2018.

Variable	n	N	%	IC95%
Nivel de precariedad				
Sin precariedad	1.013	794.746	1,96	1,77-2,16
Baja	10.107	8.363.991	20,60	20,0-21,3
Media	9.774	7.822.316	19,30	18,7-19,9
Alta	30.450	23.573.055	58,10	57,3-58,9
Grupos de edad (años)				
12-29	15.565	12.311.075	30,40	29,8-30,9
30-59	30.267	24.100.921	59,40	58,9-60,0
60 y más	5.512	4.142.112	10,20	9,82-10,6
Sexo				
Hombres	30.145	23.550.871	58,10	41,4-42,4
Mujeres	21.199	17.003.237	41,90	57,6-58,6
Unión				
Unido	31.577	24.618.555	60,70	60,0-61,4
Separado	6.820	5.385.320	13,30	12,9-13,7
Soltero	12.947	10.550.233	26,00	25,4-26,6
Nivel educativo				
Sin estudios	2.154	1.566.628	3,86	3,61-4,14
Educación básica	29.277	22.638.068	55,80	55,1-56,6
Educación media superior	10.878	8.838.245	21,80	21,2-22,4
Superior y más	9.035	7.511.167	18,50	17,9-19,2
Tipo de familia (o arreglos residenciales)				
Unipersonal	2.906	2.296.097	5,66	5,37-5,97
Nuclear	31.008	24.503.861	60,40	59,5-61,3
Extendida	11.799	9.345.363	23,00	22,3-23,8
Corresidencia	5.631	4.408.787	10,90	10,3-11,5
Conteo de discapacidades				
0	36.032	28.661.228	70,70	70,0-71,3
1	8.950	7.050.770	17,40	16,9-17,9
2 a 8	6.362	4.842.110	11,90	11,5-12,4
Variable	n	N	Media	DE
Activos en el hogar	51.344	40.554.108	0	1

DE: desviación estándar; IC95%: intervalo de 95% de confianza.

Fuente: elaboración propia con base en los microdatos de la ENSANUT 2018 ^23^.

Nota: estratos = 328; PSU’s (*primary sample units*) = 6,014; VCE (*Variance-Covariance Matrix of Estimators*) = linealizado.

Por otro lado, el 55,8% cuentan con educación básica y cerca del 60% de ellos habitan en hogares con arreglos nucleares, es decir compuestos por cónyuges y/o hijos. Es interesante que aproximadamente el 70% de los casos no presentan alguna discapacidad, y la proporción de personas afectadas entre 2 a 8 limitaciones funcionales es apenas mayor al 10%. 

**Figura 1:**
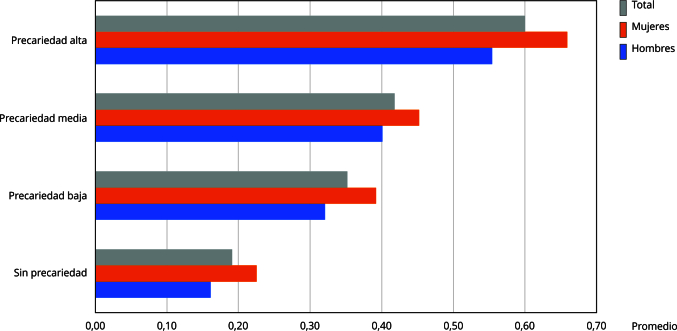
Promedio del número de limitaciones funcionales por nivel de precariedad. *Encuesta Nacional de Salud y Nutrición* (ENSANUT), México, 2018.

La Figura 1 muestra cómo el promedio de limitaciones va aumentando conforme sube el nivel de precariedad del trabajo actual. Además de que las mujeres tienen una mayor presencia de limitaciones en todos los niveles de precariedad. Esta relación muestra una relación entre las condiciones precarias del trabajo actual y las limitaciones funcionales de la población.

Fuente: elaboración propia con base en los microdatos de la ENSANUT 2018 ^23^.

La Tabla 2 muestra los coeficientes para el componente binario (la propensión entre no tener alguna limitación y tener al menos una) de los diferentes modelos ajustados. Como puede observarse, en todos los niveles de precariedad, los coeficientes tienden a disminuir conforme se avanza en la integración de covariables (modelos 1 a 7), lo que indica que la asociación entre el nivel de precariedad y limitaciones funcionales se atenúa. No obstante, el nivel de precariedad del trabajo mantiene una asociación estadísticamente significativa en el nivel bajo (β = 0,398; p < 0,05) y alto (β = 0,635; p < 0,001) lo que implica que las condiciones precarias del trabajo afectan a la propensión a sufrir una limitación funcional (modelo 7). 

**Tabla 2:** Componente binario del modelo *hurdle* con aproximación sucesiva de variables. *Encuesta Nacional de Salud y Nutrición* (ENSANUT), México, 2018.

Binario	1	2	3	4	5	6	7
Nivel de precariedad							
Baja	0,425	0,432	0,470 *	0,413 **	0,399 **	0,398 **	0,398 **
Media	0,600 *	0,621 *	0,541 *	0,375 **	0,359	0,356	0,354
Alta	1,101 ***	1,108 ***	0,879 ***	0,656 ***	0,643 ***	0,638 ***	0,635 ***
Sexo							
Mujeres		0,113 *	0,166 ***	0,178 ***	0,139 ***	0,139 ***	0,140 ***
Grupos de edad (años)							
30-59			0,672 ***	0,581 ***	0,572 ***	0,566 ***	0,568 ***
60 y más			1,520 ***	1,350 ***	1,319 ***	1,301 ***	1,303 ***
Nivel educativo							
Educación básica				-0,266 ***	-0,259 ***	-0,252 ***	-0,248 ***
Educación media superior				-0,548 ***	-0,540 ***	-0,526 ***	-0,519 ***
Superior y más				-0,662 ***	-0,646 ***	-0,633 ***	-0,623 ***
Unión							
Separado					0,176 ***	0,130 **	0,130 **
Soltero					0,030	-0,016	-0,016
Tipo de familia							
Nuclear						-0,121	-0,118
Extendida						0,015	0,019
Corresidencia						0,068	0,072
Activos en el hogar							-0,006
n	51.344						
N	40.554.108						
Estratos	328						
PSU’s	6,014						

PSU’s: *primary sample units*.

Fuente: elaboración propia con base en los microdatos de la ENSANUT 2018 ^23^.

* p < 0,01;

** p < 0,05;

*** p < 0,001.

Asimismo, los resultados del modelo completamente ajustado (modelo 7, Tabla 2) indican que el sexo, el grupo de edad y el nivel educativo tienen inclinación a asociarse con la presencia de limitaciones funcionales de manera significativa, por un lado, ser mujer (β = 0,140; p < 0,001); tener 60 años o más (β = 1,303; p < 0,001) y estar separado(a) (β = 0,130; p < 0,05) aumentan la proclividad hacia presentar al menos una limitación funcional. Además, conforme se aumenta el nivel educativo disminuye la tendencia a presentar alguna limitación, dicha asociación se mantuvo significativa en todos los niveles educativos con una disminución de β = -0,248 entre quienes cuentan con educación básica frente a los que no cuentan algún tipo de educación; de β = -0,519) entre quienes tienen educación media superior frente a quienes no cuentan con algún tipo de educación; y una disminución de β = -0,623 entre los que cuentan con nivel superior y posgrado con respecto a los que no tienen ninguna formación académica. En cambio, el índice de activos en el hogar, y el tipo de arreglo residencial dentro del hogar no tienen relaciones significativas.

La Tabla 3 muestra la asociación que tiene cada variable en el aumento de las limitaciones funcionales. Es interesante que el nivel de precariedad laboral (β_bajo_ = 0,335; p < 0,001; β_medio_ = 0,356; p < 0,001: β_alto_ = 0,473; p < 0,001), el sexo (β_mujer_ = 0,129; p < 0,001) y la edad (β_30-59 años_ = 0,554; p < 0,001; β_60 y más_ = 1,339; p < 0,001), se asoció positivamente con el aumento en el número de discapacidades, aún después de controlar por las demás covariables (modelo 7). Por otro lado, el nivel educativo (β_educación básica_ = -0,246; p < 0,001; β_educación media superior_ = -0,367; p < 0,001; β_educación superior y posgrado_ = -0,408; p < 0,001), estar soltero (β =-0,091; p < 0,001), y cualquier arreglo residencial distinto a las familias unipersonales (β_nuclear_ = -0,239; p < 0,001; β_extendida_ = -0,261; p < 0,001; β_corresidencia_ = -0,264, p < 0,001) disminuyeron la propensión a aumentar el número de alguna limitación funcional.

**Tabla 3:** Componente conteo modelo *hurdle* con aproximación sucesiva de variables. *Encuesta Nacional de Salud y Nutrición* (ENSANUT), México, 2018.

Conteo	1	2	3	4	5	6	7
Nivel de precariedad							
Baja	0,332 *	0,337 *	0,357 *	0,340 *	0,337 *	0,335 *	0,335 *
Media	0,423 *	0,441 *	0,420 *	0,359 *	0,358 *	0,356 *	0,356 *
Alta	0,592 *	0,597 *	0,557 *	0,467 *	0,471 *	0,474 *	0,473 *
Sexo							
Mujeres		0,116 *	0,124 *	0,134 *	0,116 *	0,129 *	0,129 *
Grupos de edad (años)							
30-59			0,642 *	0,609 *	0,571 *	0,553 *	0,554 *
60 y más			1,506 *	1,430 *	1,373 *	1,339 *	1,339 *
Nivel educativo							
Educación básica				-0,253 *	-0,251 *	-0,247 *	-0,246 *
Educación media superior				-0,378 *	-0,370 *	-0,368 *	-0,367 *
Superior y más				-0,418 *	-0,402 *	-0,409 *	-0,408 *
Unión							
Separado					0,123 *	0,071 **	0,071 **
Soltero					-0,061 ***	-0,091 *	-0,091 *
Tipo de familia							
Nuclear						-0,239 *	-0,239 *
Extendida						-0,262 *	-0,261 *
Corresidencia						-0,264 *	-0,264 *
Activos en el hogar							-0,001
n	51.344						
N	40.554.108						
Estratos	328						
PSU’s	6,014						

PSU’s: *primary sample units.*

Fuente: elaboración propia con base en los microdatos de la ENSANUT 2018 ^23^.

* p < 0,001;

** p < 0,05;

*** p < 0,01.

La Tabla S2 del Material Suplementario (https://cadernos.ensp.fiocruz.br/static//arquivo/suppl-e00102124_8865.pdf) muestra los marginales predichos para el modelo completo que controla por las covariables. Los valores de los marginales se pueden interpretar de manera directa y permiten conocer el sentido y dirección de la asociación entre precariedad y presencia de limitaciones. 

**Figura 2:**
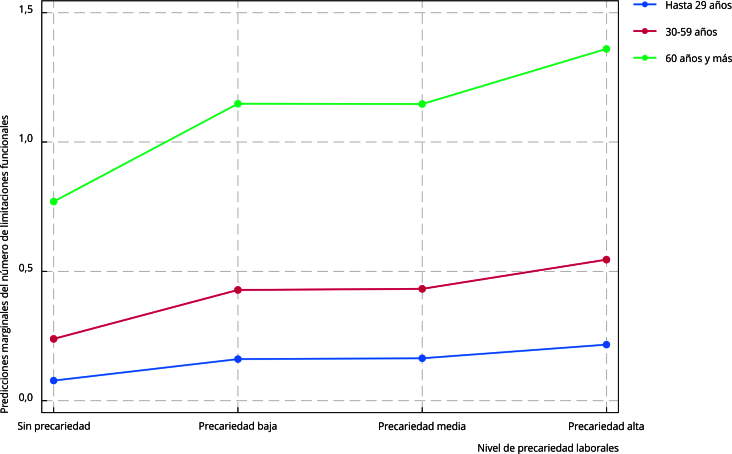
Predicciones marginales del número de limitaciones funcionales por nivel de precariedad laboral y grupo de edad. *Encuesta Nacional de Salud y Nutrición* (ENSANUT), México, 2018.

La Figura 2 representa las predicciones marginales del número de limitaciones funcionales, dependiendo de: (1) el nivel de precariedad del trabajo actual; y (2) los grupos de edad. Es interesante notar que conforme aumenta el nivel de precariedad laboral, también lo hacen las predicciones de limitaciones, en todos los grupos de edad. No obstante, esta tendencia hacia el aumento en las limitaciones funcionales es mucho más marcada para las personas adultas mayores (60 años y más).

Fuente: elaboración propia con base en los microdatos de la ENSANUT 2018 ^23^.

## Discusión

A partir de los datos de una encuesta nacional de salud a nivel poblacional de México, este estudio encontró que un mayor nivel de precariedad laboral no solo aumenta la probabilidad de presentar al menos una limitación funcional, sino que también se asocia con un mayor número de limitaciones funcionales. 

Los resultados concuerdan con diferentes investigaciones donde consideran que diferentes tipos de trabajo tienen una mayor propensión hacia las limitaciones ^1^ ^,^ ^2^, no obstante, es necesario aclarar que no es el tipo de trabajo lo que se asocia con las limitaciones en sentido estricto, sino con la desprotección de las condiciones de trabajo en general que padecen los trabajadores, por lo que se deben proponer nuevas y mejores maneras de medir la precariedad sobre todo en entornos en donde el trabajo remoto es más común y en donde la flexibilidad laboral se encuentra también diferenciada.

Las aproximaciones teóricas más importantes consideran que la relación entre el trabajo y la salud depende en gran medida de una serie de interacciones entre una gama de factores como el estrés y la privación de recursos materiales. En general, se considera que estas asociaciones pueden ser directas e indirectas ^16^, y sus relaciones son distintas en varios niveles ^15^. Por un lado, el empleo precario conlleva una carga de incertidumbre que puede afectar la salud a través de factores psicosociales como el estrés, el clima laboral, y la falta de integración social. Asimismo, aspectos como el ingreso, las políticas laborales específicas y la capacidad adquisitiva son decisivas no solamente en la tranquilidad de los trabajadores, sino en el tiempo disponible que tienen para actividades de dispersión, e inclusive en la capacidad de gestión que tienen de este para atender sus necesidades físicas, y sociales.

Lo primero que llama la atención es que cerca de un tercio de los entrevistados son jóvenes, por lo que es posible que haya una subrepresentación de los efectos del trabajo precario en la salud, debido a que la población recién ingresa al mercado laboral. Asimismo, es interesante que ser mujer se relacione de manera positiva con un mayor número de discapacidades, es probable que las mujeres trabajadoras, tengan que cumplir con una doble jornada laboral, por un lado, asisten a sus trabajos de rutina, y al mismo tiempo regresan a casa a atender las labores de reproducción del hogar ^32^ ^,^ ^33^ ^,^ ^34^ ^,^ ^35^ y el cuidado de la familia ^36^. Esto implica la generación de conflictos entre las ocupaciones familiares y laborales ^37^, lo que puede desencadenar problemas de salud física y mental ^38^.

Por otro lado, los procesos de separación tienen efectivamente una relación con el aumento de las limitaciones funcionales, aunque no necesariamente aumentan la propensión a tener al menos una limitación. Algunas hipótesis que parecen adecuarse a los resultados, como que los cónyuges tienen un papel importante en el diagnóstico de algunos problemas e inclusive pueden actuar de manera cooperativa para la reducción de hábitos poco saludables ^39^ ^,^ ^40^.

El nivel educativo actúa como un factor protector ante el aumento de discapacidades, esto es debido a que los trabajadores con mayor preparación académica tienen más elementos para buscar atención especializada en etapas tempranas de la alteración de sus capacidades ^41^, o tomar decisiones asertivas sobre su estilo de vida en favor de evitar la exposición a riesgos ^42^ ^,^ ^43^.

En general, la relación más fuerte e importante es la edad, en donde el curso de vida de las personas los ha hecho acumular una serie de desventajas en varias dimensiones, lo que provoca que se mantengan trabajando y experimenten un mayor número de limitaciones funcionales en su día a día. Asimismo, es importante hacer hincapié en que ser mujer tiene una fuerte relación con el aumento en limitaciones, por lo que es necesario promover el uso de mejores instrumentos para analizar el trabajo doméstico y las dobles jornadas laborales, que pueden ser la causa de su mayor propensión al aumento de discapacidades.

### Fortalezas y limitaciones

Como fortaleza, este análisis se realizó con una muestra representativa a nivel nacional de la población de México. Otro punto fuerte de este estudio es el hecho de que adoptó un enfoque integral de la ocupación al aproximarse a la precariedad laboral en lugar de centrarse en estatus laboral o tipo de ocupación. 

Debido a la naturaleza transversal de los resultados, la relación entre precariedad y limitaciones está sujeta a causalidad inversa. Se entiende que en la medida en que se aumente el nivel de precariedad también se aumenta la propensión a las limitaciones funcionales, no obstante, puede haber mecanismos que reproduzcan las desigualdades entre aquellas personas que desarrollaron limitaciones antes de la entrada al trabajo, y cuya condición los privara de acceder a posiciones no precarias, reproduciendo así su condición de precariedad. Desafortunadamente, la información se recolecta por autorreporte por lo que se basa en la memoria de los encuestados y puede estar sujeta a errores de reporte. 

Algunas de las limitaciones más importantes tienen que ver con la disposición y operacionalización de las variables. El análisis del estado de funcionalidad de las personas tiene diferentes aproximaciones: por un lado, una línea de investigación se basa en el supuesto teórico de que la discapacidad y la salud son parte de un *continuum* ^22^ ^,^ ^44^. Otras aproximaciones se decantan por definir la discapacidad de manera dicotómica, considerando que cualquier dificultad o afectación en alguna dimensión de funciones básicas debe de ser catalogada como discapacidad ^2^ ^,^ ^45^. 

Con respecto a la medición de la precariedad es necesario recalcar que diferentes investigaciones se han enfocado en calcular un indicador que permita sintetizar la complejidad (en términos multinivel y multidimensionales) de la precariedad ^46^, algunos trabajos lo han intentado a través de una reducción de dimensionalidad en términos multivariados ^6^, aunque sostienen que la mejor solución para medir la precariedad no es estadística sino teórica ^6^ ^,^ ^11^. Lamentablemente, la ENSANUT no cuenta con una pregunta que permita saber si los trabajadores tienen contrato o no. Ese indicador, aunque relevante, no es imprescindible. En este sentido, no hace falta tener un indicador explícito del nivel de inseguridad laboral, dado que en general sus efectos y características están muy asociados con las condiciones de desprotección del trabajo ^6^. 

Hay investigaciones que no estudian todas las dimensiones analíticas ^11^ y, aun así, gracias a la fuerte relación teórica entre los indicadores pueden hacer uso del concepto de precariedad laboral, aunque se debe de tener en cuenta que los resultados pueden estar sub representando los efectos de la precariedad en la salud. Por otro lado, todas las personas que respondieron que trabajan sin pago, se clasificaron con ausencia de las prestaciones laborales, y debido a que la ENSANUT no tiene como propósito medir a fondo las características de las ocupaciones, es imposible conocer el nivel de informalidad en los trabajos, por lo que puede estar subestimado. 

## Conclusiones

Los hallazgos de esta investigación refuerzan la idea de que diversos aspectos de la vida laboral influyen directamente en la salud, en particular en la funcionalidad. Este estudio evidencia la relación entre la precariedad laboral y la presencia de limitaciones funcionales, tanto en su presencia como en su magnitud.

Debido a que los trabajadores enfrentan un contexto de incertidumbre laboral en México, caracterizado por la desregulación y la flexibilidad en las relaciones laborales, es fundamental garantizar que las personas accedan a empleos dignos. Además, se deben implementar políticas que no solo reduzcan la precariedad, sino que también prevengan la reproducción de desigualdades estructurales en el ámbito laboral, las cuales pueden contribuir al desarrollo de limitaciones funcionales.

Entre las recomendaciones que podrían derivarse del estudio, se sugiere la implementación de políticas laborales que reduzcan la inestabilidad y mejoren las condiciones de empleo, por ejemplo: implementar mecanismos de inspección y regulación efectivos de los trabajos, con el fin de prevenir efectos adversos en la salud funcional. Asimismo, se plantea la necesidad de universalizar los programas de protección social que garanticen el acceso a atención médica y servicios de rehabilitación para trabajadores en situación de precariedad o con limitaciones funcionales derivadas de condiciones laborales adversas. 

Se destaca la importancia de desarrollar programas de prevención y monitoreo de la salud ocupacional que identifiquen tempranamente riesgos de deterioro funcional entre los trabajadores con condiciones precarias. Estos programas podrían articularse con políticas o estrategias de salud activa en el ámbito laboral, promoviendo entornos inclusivos y ajustes razonables en trabajadores con limitaciones físicas. Finalmente, se sugiere un enfoque diferenciado por grupo etario, dado que los resultados del estudio evidenciaron patrones específicos de vulnerabilidad conforme la edad avanza.
