# Supplementary material for: Precarious employment and functional limitations: a cross-sectional analysis for the Mexican population
Source: Cad Saude Publica. 2025 Jul 4;41(6):e00102124. [Article in Spanish] doi: 10.1590/0102-311XES102124 (PMC12236084; doi:10.1590/0102-311XES102124)
Supplement: Supplementary file 1 [file 1678-4464-csp-41-06-ES102124-s.pdf]

# Material Suplementario

**Tabla S1** Descripción de los indicadores de desprotección de las condiciones de trabajo.

| INDICADOR | DEFINICIÓN OPERACIONAL |
|---|---|
| Seguridad social | Evalúa la posibilidad de recibir atención médica por parte del IMSS, ISSSTE u otro servicio privado |
| Aguinaldo | Evalúa la posibilidad de recibir el pago de al menos 15 días de salario después de un año de servicio. En México se paga antes del 20 de diciembre de cada año |
| Vacaciones | Mide la posibilidad de contar con un periodo de descanso anual y pagado |
| Utilidades | En el cuestionario de la ENSANUT se pregunta si reciben o puede recibir reparto de utilidades o prima vacacional. En general se considera que el reparto de utilidades es el derecho a recibir parte de las ganancias de una empresa o patrón, cuyo monto se determina de acuerdo con el tiempo de trabajo y las utilidades del centro de trabajo |
| Retiro | Indica si los trabajadores cuentan con un servicio de fondo de ahorro para el retiro, es decir, busca conocer si se están acumulando recursos en cuentas individuales de manera que los trabajadores puedan disponer de pensiones al concluir su vida laboral |
| Crédito | Mide la posibilidad de acceder a un crédito para la vivienda similar a Infonavit o Fovisste |
| Guardería | Evalúa la posibilidad de que las personas trabajadoras con hijos tengan acceso a una guardería durante sus horas de trabajo |
| Maternidad/Paternidad | Mide la posibilidad de contar con una licencia para ausentarse del trabajo antes y/o después del parto/ adopción |
| Seguro de vida | Establece la tenencia o no de un instrumento financiero por el que se otorga dinero en caso de que los trabajadores mueran |
| Prestamos | Mide la posibilidad de contar con acceso a un préstamo personal o caja de ahorro con la finalidad de contar con recursos financieros de manera inmediata |

Fuente: elaboración própria.

**Tabla S2** Marginales predichos del modelo completo. *Encuesta Nacional de Salud y Nutrición* (ENSANUT), México, 2018.

| **Variable** | **dy/dx** | **Error estándar** | **z** | **P > \|z\|** |
|---|---|---|---|---|
| Nivel de precariedad | | | | |
| Baja | 0.179 | 0.032 | 5.590 | 0.000 |
| Media | 0.182 | 0.031 | 5.880 | 0.000 |
| Alta | 0.288 | 0.031 | 9.200 | 0.000 |
| Sexo | | | | |
| Mujeres | 0.090 | 0.011 | 8.550 | 0.000 |
| Grupos de edad | | | | |
| 30-59 | 0.305 | 0.011 | 26.680 | 0.000 |
| 60 y más | 1.073 | 0.033 | 32.860 | 0.000 |
| Nivel educativo | | | | |
| Educación básica | -0.197 | 0.033 | -5.970 | 0.000 |
| Educación media superior | -0.303 | 0.036 | -8.390 | 0.000 |
| Superior y más | -0.336 | 0.038 | -8.930 | 0.000 |
| Unión | | | | |
| Separado | 0.061 | 0.018 | 3.490 | 0.000 |
| Soltero | -0.047 | 0.016 | -2.930 | 0.003 |
| Tipo de familia | | | | |
| Nuclear | -0.148 | 0.028 | -5.360 | 0.000 |
| Extendida | -0.136 | 0.028 | -4.760 | 0.000 |
| Corresidencia | -0.128 | 0.033 | -3.910 | 0.000 |
| Activos en el hogar | -0.001 | 0.007 | -0.210 | 0.835 |

Nota: los resultados muestran que en la medida en que aumentan las condiciones de precariedad del trabajo actual se tiene una mayor propensión a aumentar el número de limitaciones funcionales ($P_{bajo}$= 0.179; $P_{medio}$=0.182: $P_{alto}$=0.288). Asimismo, estas limitaciones se van acentuando conforme se avanza en el curso de vida, es decir que pertenecer a la cohorte de edad de más de 60 años hace a las personas más propensas a tener al menos una discapacidad (P=1.073). Asimismo, las mujeres (P=0.090) y los separados (P=0.061) tienen mayor inclinación a presentar limitaciones.

Por otro lado, hay una relación negativa significativa entre el nivel educativo ($P_{educación\ básica}$= -0.197; $P_{educación\ media\ superior}$= -0.303; $P_{educación\ superior\ y\ posgrado}$ = -0.336), el tipo de arreglo residencial ($P_{nuclear}$= -0.148; $P_{extendida}$= -0.136; $P_{corresidencia}$= -0.128) y estar soltero (P= -0.047) en la presencia de limitaciones. Esto quiere decir que en la medida en que el nivel educativo sube, se reducen las probabilidades de presentar limitaciones, lo que también sucede con todos los tipos de arreglos residenciales.

Es interesante que las asociaciones positivas más fuertes son: 1) tener más de 60 años (P=1.073); 2) nivel de precariedad alta (P=0.288). En contraposición, las relaciones negativas con coeficientes más altos son del nivel educativo ($P_{educación\ superior\ y\ posgrado}$= -0.336).

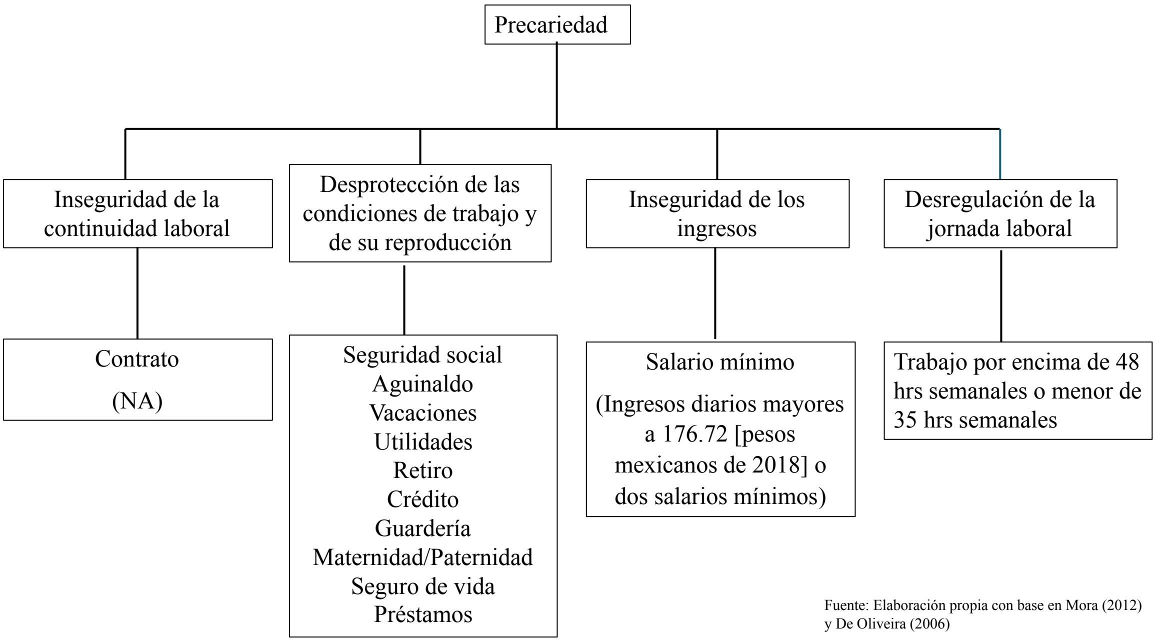


**Figura S1** Dimensiones teóricas de la precariedad laboral.

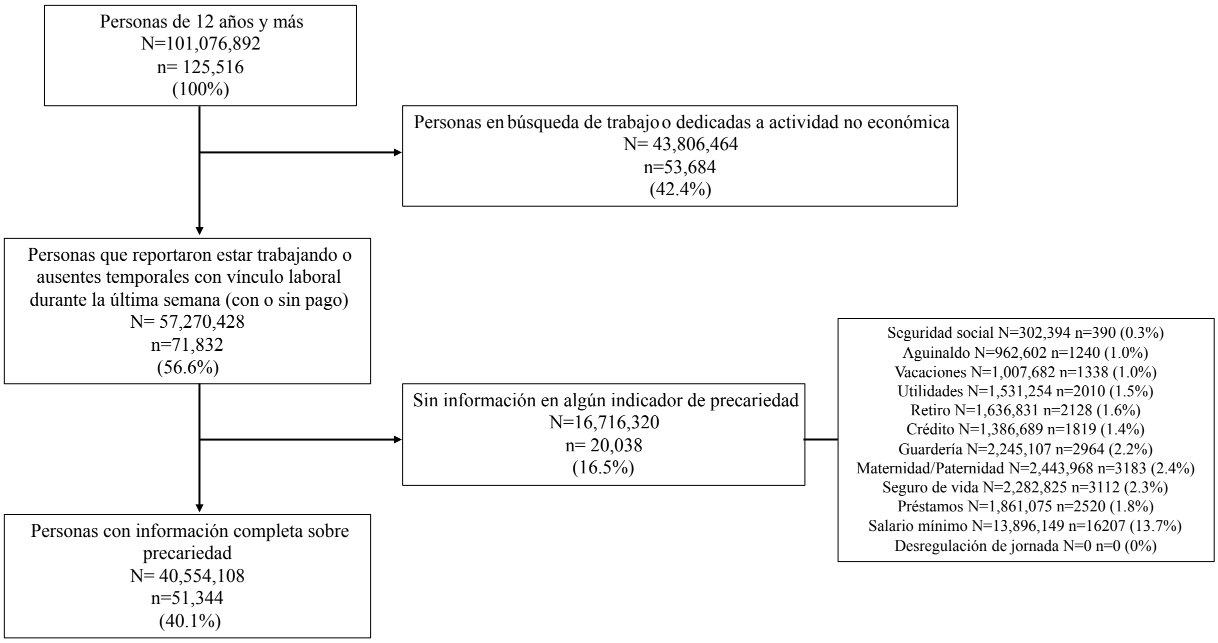


Fuente: Elaboración propia con base en microdatos de la ENSANUT 2018

**Figura S2** Selección de la muestra. *Encuesta Nacional de Salud y Nutrición* (ENSANUT), México, 2018.

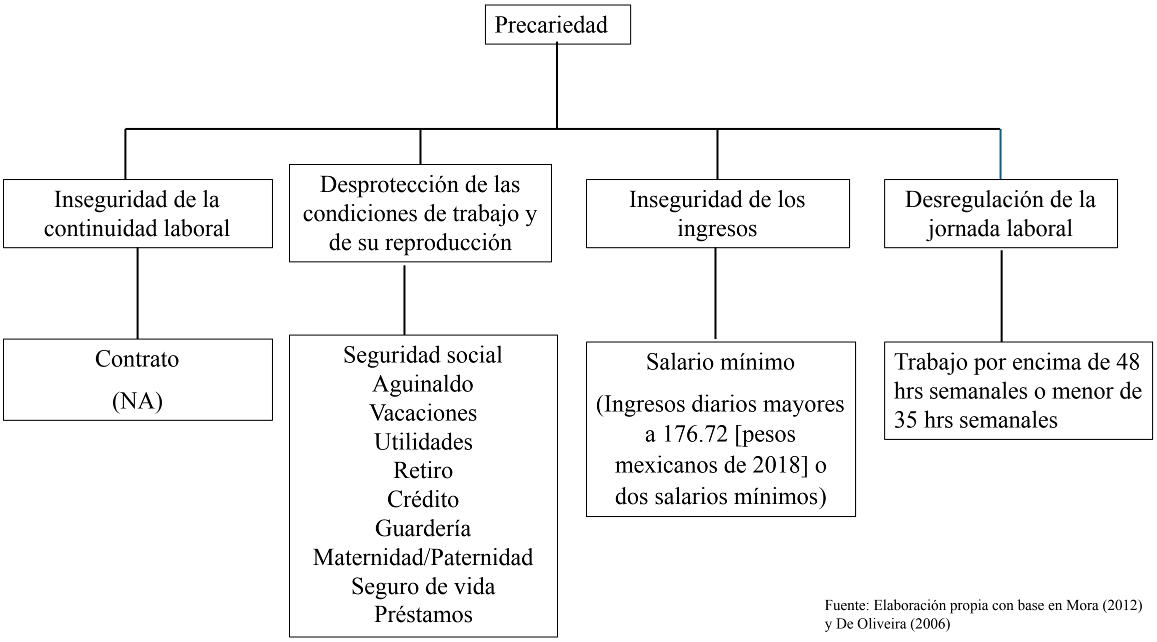


**Figura S3** Indicadores de las dimensiones de precariedad laboral utilizadas a partir de datos *Encuesta Nacional de Salud y Nutrición* (ENSANUT), México, 2018.
